# Spatial Structure and Activity of Synthetic Fragments of Lynx1 and of Nicotinic Receptor Loop C Models

**DOI:** 10.3390/biom11010001

**Published:** 2020-12-22

**Authors:** Konstantin S. Mineev, Elena V. Kryukova, Igor E. Kasheverov, Natalia S. Egorova, Maxim N. Zhmak, Igor A. Ivanov, Dmitry A. Senko, Alexey V. Feofanov, Anastasia A. Ignatova, Alexander S. Arseniev, Yuri N. Utkin, Victor I. Tsetlin

**Affiliations:** 1 Shemyakin-Ovchinnikov Institute of Bioorganic Chemistry, Russian Academy of Sciences, 117997 Moscow, Russia; evkr@mail.ru (E.V.K.); shak_ever@yahoo.com (I.E.K.); natalyegorov@yandex.ru (N.S.E.); mzhmak@gmail.com (M.N.Z.); chai.mail0@gmail.com (I.A.I.); senko-da@yandex.ru (D.A.S.); avfeofanov@yandex.ru (A.V.F.); aignatova_83@mail.ru (A.A.I.); aars@nmr.ru (A.S.A.); yutkin@yandex.ru (Y.N.U.); victortsetlin3f@gmail.com (V.I.T.); 2 Department of Physico-Chemical Biology and Biotechnology, Moscow Institute of Physics and Technology, 141700 Dolgoprudnyi, Russia; 3 Laboratory of Molecular Biology and Biochemistry, Institute of Molecular Medicine, Biomedical Science and Technology Park, Sechenov First Moscow State Medical University, 119991 Moscow, Russia; 4 Department of Chemistry, Lomonosov Moscow State University, 119991 Moscow, Russia; 5 Biological Faculty, Lomonosov Moscow State University, 119991 Moscow, Russia; 6 Institute for Physics and Engineering in Biomedicine, National Research Nuclear University MEPhI, 115409 Moscow, Russia

**Keywords:** nicotinic acetylcholine receptors, three-finger proteins, peptide fragments, spatial structure, nuclear magnetic resonance, circular dichroism, radioligand assay

## Abstract

Lynx1, membrane-bound protein co-localized with the nicotinic acetylcholine receptors (nAChRs) and regulates their function, is a three-finger protein (TFP) made of three β-structural loops, similarly to snake venom α-neurotoxin TFPs. Since the central loop II of α-neurotoxins is involved in binding to nAChRs, we have recently synthesized the fragments of Lynx1 central loop, including those with the disulfide between Cys residues introduced at N- and C-termini, some of them inhibiting muscle-type nAChR similarly to the whole-size water-soluble Lynx1 (ws-Lynx1). Literature shows that the main fragment interacting with TFPs is the C-loop of both nAChRs and acetylcholine binding proteins (AChBPs) while some ligand-binding capacity is preserved by analogs of this loop, for example, by high-affinity peptide HAP. Here we analyzed the structural organization of these peptide models of ligands and receptors and its role in binding. Thus, fragments of Lynx1 loop II, loop C from the *Lymnaea stagnalis* AChBP and HAP were synthesized in linear and Cys-cyclized forms and structurally (CD and NMR) and functionally (radioligand assay on *Torpedo* nAChR) characterized. Connecting the C- and N-termini by disulfide in the ws-Lynx1 fragment stabilized its conformation which became similar to the loop II within the ^1^H-NMR structure of ws-Lynx1, the activity being higher than for starting linear fragment but lower than for peptide with free cysteines. Introduced disulfides did not considerably change the structure of HAP and of loop C fragments, the former preserving high affinity for α-bungarotoxin, while, surprisingly, no binding was detected with loop C and its analogs.

## 1. Introduction

α-Neurotoxins from the snake venoms having a three-loop (or three-finger) spatial structure (three-finger proteins, TFPs) are well-recognized tools in the research on the nicotinic acetylcholine receptors (nAChRs) [1,2,3]. In humans and other organisms, there are also TFPs of the Ly6 family: these proteins are either water-soluble or attached by the glycosylphosphatidyinositol anchor to the membranes and some of them are targeting distinct nAChRs subtypes, thus affecting their multiple physiological functions [4,5,6]. According to the biochemical data, NMR, cryo-electron microscopy and X-ray structures, the central loop II in TFPs plays an important role in binding to nAChRs, while the loop C contributes much to the binding from the side of the receptor [1,7,8,9]. Synthetic peptides covering a part of loop II in neurotoxins have a sufficiently high affinity for the nAChRs indicating their possible utility in drug design [7]. Recently a similar activity in the recognition of distinct nAChR subtypes was demonstrated for the first time for the loop II synthetic fragments of several TFPs of the Ly6 family, where Cys residues were added at the N-and C-termini and were left free or connected by disulfide [10]. What concerns the nAChR synthetic fragments, the capacity to bind the snake venom TFPs was earlier shown for the fragments of α1 and α7 subunits encompassing the loop C and for combinatorial peptides homologous to its part [11,12,13,14,15]. Interestingly, one of the latter, so-called high-affinity peptide (HAP) could bind α-bungarotoxin (α-Bgt) with the same nanomolar affinity as the whole-size muscle-type nAChR [15]. Structural studies of HAP or its variants have shown that in complexes with α-Bgt these linear peptides acquire an ordered form, similar to the C-loop structures in the nAChRs or AChBPs [16,17,18,19,20], that may explain their high affinity for the toxin. However, there is still no complete clarity as to the dependence of the functional activity of peptide fragments of both TFPs and loop C from nAChRs or AChBPs on formation of secondary structure elements, leaving a hypothetical solution of this question for computer simulation and for further structural studies.

Until the publication in 2020 of the cryo-electron microscopy structure of a native *Torpedo californica* ray nAChR in complex with α-Bgt [18], there was no spatial structure of the whole-size nAChR in complex with any TFP, but the X-ray and NMR structures of the snake-venom TFPs associated with the synthetic fragments of the α1 subunit [14,21,22], with the ligand-binding domains of the α1, α7 and α9 subunits [19,23,24] and with AChBP [20] were available. Interestingly, the X-ray structure of the loop C in AChBP was used to model the TFP binding to nAChRs, although this loop was not synthesized and its binding to TFPs was not analyzed [25].

In the present communication, we carried out the structural studies (CD spectra and NMR data) of several synthetic peptides, corresponding to the loop II of the Ly6 protein Lynx1, the loop C of *Lymnaea stagnalis* AChBP and peptide HAP in a linear form and cyclized by disulfide bridge between additional flanking cysteines.

Lynx1 belongs to the family of Ly6 proteins where their protein part is structurally similar to the snake venom “three-finger” toxins containing 3 β-structural loops with the overall structure stabilized by 5 disulfides. Some of these proteins are secreted, but like many other members of this family, Lynx1 is attached to the membrane by a glycosylphosphatidyl inositol (GPI) tail [1,2]. The earlier work was focused on the Lynx1 heterologously expressed in *E. coli* and built of 74 amino acid residues, not having a GPI tail, and designated as ws-Lynx1, water-soluble Lynx1 [26]. In our recent work, keeping in mind relevant research on the activity of synthetic fragments of snake venom α-neurotoxins, we synthesized central loops of several Ly6 proteins, including that of ws-Lynx1, connected their N-and C-termini by the attached disulfide, and analyzed the activity against the muscle-type and several neuronal nAChRs [10]. Since the most similar affinity between the whole protein and its fragment was achieved in the case of ws-Lynx on binding to the *Torpedo californica* nAChR, in the present communication we analyzed by ^1^H-NMR the spatial structure of this peptide and compared it with the structure of the central loop within the whole structure of ws-Lynx1. The activity and conformation of a similar peptide with the Cys residues introduced at the N-and C-termini, but not closed into a disulfide, were also studied. In addition, since the competition with α-bungarotoxin reflects binding of the ws-Lynx1 and of its fragments to the orthosteric sites of the nAChRs and of its models, in the present work we also checked whether the introduction of a disulfide, connecting the N-and C-termini, to such peptides as combinatorial peptide HAP or fragment of the C-loop of the AChBP, resembling the nAChR orthosteric sites, influences their binding characteristics.

Thus, we were interested what effects might be introduced by a fixing disulfide on the structure and activity of the fragments both of the cholinergic ligand and of the ligand-binding site. It was found that the linear form of the synthetic fragments of TFPs of the Ly6 family is disordered, while the disulfide-cyclized form has a more stable conformation and is very similar to the respective portion of loop II within the ws-Lynx1 [26]. However, this property does not strongly affect the ability of both forms to bind to the nAChR from *T. californica*. The disulfide in loop C of AChBP had slightly increased the stability of the peptide but no ordered structure and no toxin binding was found. The additional disulfide did not strongly change a quite high secondary structure in HAP and did not affect its high affinity for α-Bgt.

## 2. Materials and Methods

### 2.1. Synthesis of Linear and Disulfide-Stabilized Fragments of Human Lynx1, of the Lymnaea Stagnalis AChBP Loop C and of the Combinatorial Peptide HAP

Linear peptides were prepared by solid-phase peptide synthesis using Fmoc/t-butyl strategy on tritylchloride-polystyrene resin (Intavis, Cologne, Germany) mostly as in [10]. Polypeptide chain assembly was performed on Syro I automatic peptide synthesizer (MultiSynTech AG, Witten, Germany). Disulfides formation was carried out under conventional oxidation conditions: prolonged incubation on air in 50% aqueous acetonitrile at room temperature in the presence N-ethyldiisopropylamine (pH 8.0). In the case of formation of vicinal disulfide in peptide *Ls*202–214^C-C^, the reaction proceeded extremely slowly with predominant formation of by-products, the best yields of target product being achieved by adding 20% dimethylsulfoxide to the peptide solution. All peptides were purified by high-performance liquid chromatography (HPLC) on a 250 × 30 mm C8 column (Dr. Maisch, Ammerbuch-Entringen, Germany) in linear gradient of acetonitrile from 10% to 40% and then lyophilized. The peptides purity was confirmed using analytical HPLC and the respective molecular masses were determined by MALDI-MS using Bruker Ultraflex I mass-spectrometer (Bruker Daltonik GmbH, Bremen, Germany) (Table 1).

### 2.2. Activity of Synthetic Fragments of Lynx1 and of Nicotinic Receptor Loop C Models in Radioligand Assay

In these studies, we used radiolabelled α-Bgt (^125^I-α-Bgt) with specific radioactivity of 500 Ci/mmol prepared as described in [27]. Competition experiments with Lynx1 fragments (compounds (1)–(3)) were performed mostly as described in [10]. Briefly, the membranes from *T. californica* ray electric organ (0.6 nM of α-bungarotoxin binding sites) were incubated in 50 μL of binding buffer (20 mM Tris-HCl buffer, pH 8.0, containing 1 mg/mL BSA) for 90 min with different concentrations of the Lynx1 peptides, followed by an additional 5-min incubation with 0.5 nM of ^125^I-α-Bgt. The membranes were applied to glass GF/C filters (Whatman, Maidstone, UK) pretreated with 0.3% polyethylenimine. The samples were then washed (3 × 4 mL) with cold 20 mM Tris-HCl buffer, pH 8.0, containing 0.1 mg/mL BSA and bound radioactivity was measured with a Wallac 1470 Wizard Gamma Counter (PerkinElmer, Waltham, MA, USA). Nonspecific ^125^I-α-Bgt binding was determined in the presence of 200-fold excess of α-cobratoxin. The results were analyzed using ORIGIN 7.5 (OriginLab, Northampton, MA, USA) fitting to one-site dose-response competition curves.

For the loop C fragments, HAP and its analogs (compounds (4)–(7)) we applied the same radioligand assay protocol, although in this case the observed decrease in binding of the ^125^I-α-Bgt to the *T. californica* nAChR is caused not by direct competition of radioligand with the studied compounds (representing in this case the receptor model), but by their interaction with the ^125^I-α-Bgt itself. In these experiments we incubated the *Torpedo* membranes (0.6 nM of α-bungarotoxin binding sites) for 180 min with 10 μM of the studied peptides, followed by an additional 5-min incubation with 0.5 nM of ^125^I-α-Bgt and similar washing and counting.

### 2.3. CD Analysis of the Synthetic Peptides

CD spectra were recorded using a JASCO J-810 spectropolarimeter (JASCO International Co., Tokyo, Japan) in the 190–250 nm spectral range in a 0.01 cm cuvette at 22 °C. Measurements were performed in the 50 mM sodium phosphate buffer (pH 7.5) at concentrations of 0.55, 0.44, 0.49, 0.68, 0.18, 0.73, 0.54, 0.59, 0.53 and 0.44 mM for compounds (1)–(10), respectively. Spectra were averaged over 4 consequent scans for each peptide. The secondary structure of peptides was calculated with the CONTIN/LL program (CDPro package) using the SMP56 protein reference set.

### 2.4. NMR Spectroscopy

For the spatial structure analysis, synthetic peptides (Ly29–43^C,C^, Ly29–43^C-C^, m*Ls*202–214^C,C^, m*Ls*202–214^C-C^) were dissolved in water (50 mM NaCl, pH 5.5). All NMR spectra were recorded at 30 °C using the Bruker Avance 700 and Bruker Avance III 600 spectrometers (Bruker Biospin Gmbh, Ettlingen, Germany), with proton working frequencies equal to 700 and 600 MHz, respectively. Complete ^1^H, ^13^C and ^15^N chemical shift NMR assignment was obtained using the set of homo- and heteronuclear NMR spectra acquired at natural abundance: 2D NOESY, ^1^H,^13^C-HSQC-TOCSY, DQF-COSY, ^1^H,^13^C-HSQC, ^1^H,^15^N-HSQC, ^1^H,^15^N-HMBC, ^1^H,^15^N-HMBC. The obtained chemical shifts were analyzed using the RCI-S^2^ software [28] to estimate the backbone mobility, SSP software to calculate the secondary structure propensities [29] and TALOS-N software to obtain the dihedral angle restraints for the relatively stable peptide Ly29–43^C-C^ [30]. Spatial structure of this peptide was calculated using the CYANA 3.98 software [31]. The obtained chemical shifts were deposited to the BMRB data base under the accession numbers 50,606 (Ly29–43^C-C^), 50,607 (Ly29–43^C,C^), 50,608 (m*Ls*202–214^C,C^) and 50,609 (m*Ls*202–214^C-C^). The data for the spatial structure of Ly29–43^C–-C^ are in a Supplementary data File S1.

To assess the backbone dynamics of the peptides, the steady-state ^1^H, ^13^C nuclear Overhauser effect (NOE) and ^13^C longitudinal relaxation rates (R1) were measured for all available Cα-H groups. Obtained values were converted to the order parameters of Cα-H vectors (S^2^) and characteristic times of internal motions τ_i_ using the formalism described in the works [32,33]. Overall rotational diffusion correlation times and hydrodynamic radii of the peptides were assessed using the Stokes-Einstein relationship from the coefficients of translational diffusion, D corrected for the peptide concentration [34]. The latter were measured by NMR using the PGSTE-watergate pulse sequence with simultaneous suppression of the convection effects and solvent signal [35].

## 3. Results

### 3.1. Synthesis of Linear and Disulfide-Stabilized Fragments of Human Lynx1 Loop II, of the L. stagnalis AChBP Loop C and of the Combinatorial Peptide HAP

All linear peptides were synthesized using the standard Fmoc-strategy. To obtain the target peptides Ly29–43^C-C^, *Ls*202–214^C-C^, m*Ls*202–214^C-C^ and HAP^C-C^, the disulfide bonds formation was performed by oxidation with air oxygen, giving a final yield of cyclized peptides at this stage from 30 to 40%, and did not cause difficulties with the exception of *Ls*202–214^C-C^. In the case of *Ls*202–214^C-C^ product, even under the optimally selected conditions, the yield of the target peptide with vicinal disulfide did not exceed 3%. According to analytical HPLC data, the purity of the obtained target peptides was >95% and their masses closely corresponded to the calculated ones (Table 1). In order to prevent the formation of disulfides in peptides Ly29–43^C,C^, m*Ls*202–214^C,C^ and HAP^C,C^, they were stored always in the lyophilized form and dissolved immediately before the experiments.

### 3.2. Activity of Synthetic Fragments of Lynx1 and of Nicotinic Receptor Loop C Models in Radioligand Assay

**Figure 1:**
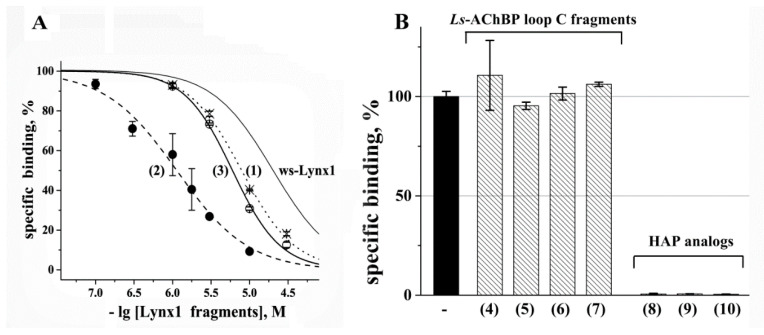
Characterization of binding activity of synthesized peptide fragments by competitive radioligand analysis using ^125^I-α-Bgt as a radioligand. (**A**) Inhibition curves for Lynx1 loop II fragments on *T. californica* nAChR. Crosses, full circles and open circles indicate the data points for compounds (1)–(3), respectively. Each point is the mean ± SEM value of two measurements for each concentration in two independent experiments. The calculated IC_50_ values (mean ± SEM) for peptides 1-3 were 7.9 ± 0.2, 1.1 ± 0.1 and 6.0 ± 0.2 μM, respectively. For comparison, the inhibition curve for ws-Lynx1 (no symbols) on the same receptor under the same conditions (IC_50_ 24 μM) is shown [26]. (**B**) Bar graph presentation of the decrease in specific binding of ^125^I-α-Bgt to *T. californica* nAChR in the presence of *L. stagnalis* AChBP loop C and HAP analogs. Concentration of peptides (4)–(10) was 10 μM at which no “inhibition” for loop C fragments (102 ± 7 % of specific binding) and complete “inhibition” for HAP analogs (0.6 ± 0.1 % of specific binding) was revealed. The specific binding in the absence of peptides is accepted as 100%. Data are presented as mean ± SEM.

The biological activity of Lynx1 fragments Ly29–43, Ly29–43^C,C^ and Ly29–43^C-C^ (peptides (1)–(3), respectively) was evaluated as their ability to compete with radioactive α-bungarotoxin (^125^I-α-Bgt) for binding to the muscle-type nAChR from *T. californica* ray electric organ. All of them displaced the radioligand from the receptor in the range of μM concentrations (Figure 1A). Herewith the linear peptide Ly29–43 and cyclized Ly29–43^C-C^ showed approximately equal inhibition efficiency (the respective IC_50_ values were 7.9 ± 0.2 and 6.0 ± 0.2 μM). Interestingly, the linear form of Lynx1 fragment 29–43 with free cysteines at N- and C- termini was the most efficient with IC_50_ = 1.1 ± 0.1 μM, with the activity considerably surpassing that of the ws-Lynx1.

The same competitive radioligand assay was used to evaluate the binding activity of *L. stagnalis* AchBP loop C and HAP analogs, although in this case, not the degree of competition between ^125^I-α-Bgt and the tested peptides for binding to the target (*T. californica* nAChR) was evaluated, but the ability of these peptides to bind the radioligand itself, thus preventing its association with the receptor. This method allowed us to demonstrate the high efficiency of HAP analogs; at 10 μM they have completely bound ^125^I-α-Bgt (Figure 1B). The effectiveness of this binding did not depend on whether HAP was in a linear (peptides (8), (9)) or cyclized form (peptide (10)). Interestingly, the native *L. stagnalis* AChBP loop C (which served as the basis for the design of HAP) in both linear and cyclized forms (peptides (4)–(7)) did not reveal any ability to bind the radioligand at the same concentration (Figure 1B).

### 3.3. CD Analysis of the Synthetic Peptides

**Figure 2:**
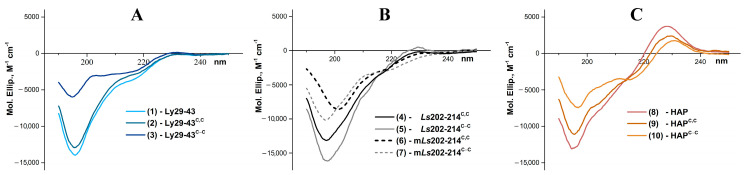
Characterization of secondary structure of peptides (1)–(10) at pH 7.5 by CD spectroscopy. (**A**) CD spectra of Lynx1 loop II fragments—Ly29–43, Ly29–43^C,C^ and Ly29–43^C-C^. (**B**) CD spectra of native or mutated *L. stagnalis* AChBP loop C—*Ls*202–214^C,C^*, Ls*202–214^C-C^*,* m*Ls*202–214^C,C^*,* m*Ls*202–214^C-C^. (**C**) CD spectra of HAP and its analogs—HAP, HAP^C,C^ and HAP^C-C^.

The obtained CD spectra and the content of secondary structure elements formally calculated from them allow us to make reliable conclusion about very low content of α-helices in the studied peptides in an aqueous solution (Table 2, Figure 2). The differences in content of other secondary structure elements, taking into account the relativity of these values for small peptides, are too small to draw certain conclusions.

### 3.4. NMR Analysis of Synthetic Lynx1 Fragments

CD spectroscopy provides a quantitative view on the peptide secondary structure; however, the results lack the atomic details. Therefore, we applied the heteronuclear NMR spectroscopy to identify such atomic differences using the linear and cyclized forms of a Lynx1 loop II fragment: Ly29–43^C,C^ and Ly29-–43^C-C^ which differed in their affinities towards *T. californica* nAChR (Figure 1A). For both peptides we obtained the full ^1^H, ^13^C and ^15^N chemical shift assignments, which were then analyzed using several approaches. As a first step, we characterized the overall compactness of the spatial structure by measuring the translational diffusion of the two peptides. The diffusion coefficient of Ly29–43^C-C^ was equal to (261 ± 2) 10^−12^ m^2^/s, corresponding to the hydrodynamic radius of 1.07 nm and molecular weight of a globular protein of 2.1 kDa [37]. Thus, Ly29–43^C-C^ is compact and behaves as a globular protein, if this term is applicable for such a small peptide. In contrast, diffusion of Ly29–43^C,C^ is much slower, (215 ± 2)·10^−12^ m^2^/s, which corresponds to 3.8 kDa. Therefore, Ly29–43^C,C^ does not form a compact spatial structure and behaves as a disordered protein.

**Figure 3:**
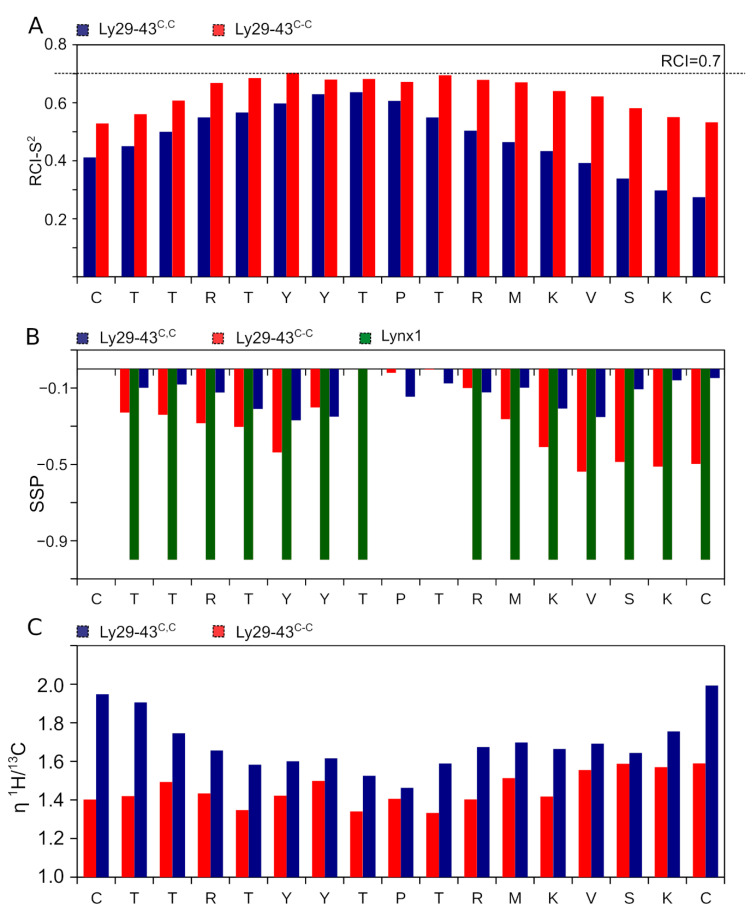
Distributions of RCI-S^2^ (**A**), SSPs (**B**) and ^1^H,^13^C steady-state NOEs (**C**) for Ly29–43^C,C^ (blue bars) and Ly29–43^C-C^ (red bars). Negative values of SSP correspond to the β-sheet or extended conformation. SSPs for the secondary structure of the homologous to Ly29–43 region of full-length human Lynx1 (PDB ID: 2L03) is shown by green bars on panel **B** for comparison. Heteronuclear NOE was measured at 30 °C and 700 MHz.

Next, we analyzed the dynamic structure of two peptides using the concepts of random-coil index (RCI) [28] and secondary structure propensity (SSP) [29]. RCI converts the chemical shifts into the backbone order parameter RCI-S^2^. Regions with RCI-S^2^ greater than 0.7 might be considered as stable and structured, while lower magnitudes of the parameter correspond to the dynamic and partially disordered backbone. Ly29–43^C,C^ is characterized by low RCI-S^2^, 0.5 on average, reaching 0.6 close to the central Pro residue (Figure 3A). In contrast, Ly29–43^C-C^ RCI-S^2^ reveals a plateau of 0.7 on the region 4–12, indicating that the cyclic peptide is substantially more stable, however, is still partially disordered.

SSP is a well-established approach to describe the structure of intrinsically disordered proteins and peptides. SSP values greater than zero indicate the propensity of the helical conformation, while the negative SSPs reveal the possibility of β-sheet or extended (in contrast to disordered) conformation to be formed in the region. SSPs of Ly29–43^C-C^ residues reveal a relatively high propensity (up to 50%) of the extended conformation in the N- and C-terminal regions with the unstructured central part of the peptide (Figure 3B). Comparison with the structure of the full size Lynx1 central loop (green bars in Figure 3B) reveals that the extended conformation occurs at the same sites, where the β-structure is observed in Lynx1.

To further characterize the structure of Ly29–43^C-C^, we applied the nuclear Overhauser effect NMR spectroscopy (NOESY). No characteristics for β-sheet backbone connectivities are observed in the NOESY NMR spectra, mainly due to the low chemical shift dispersion. However, two long-range side-chain contacts are seen that support the transient β-hairpin structure of Ly29–43^C-C^ (between the Y7 aromatic ring and V14 methyl groups and between the Y6 ring and K13 methylenes). All aforesaid implies that at least part of the time, Ly29–43^C-C^ resides in the β-hairpin conformation, close to structure of the homologous region of the parent Lynx1 protein. In contract, Ly29–43^C,C^ reveals the small 10–20% propensity for the extended conformation on the whole length of the peptide, which is far from the structure of this region within the full-length Lynx1.

**Figure 4:**
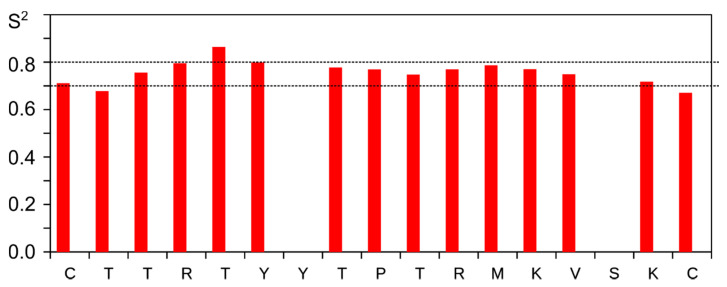
Distribution of C-H order parameters (S^2^) obtained for Ly29–43^C-C^.

Last, we employed the NMR relaxation parameters of α-carbons to assess the intramolecular mobility of the peptides’ backbone. Two parameters, ^1^H-^13^C steady-state NOE (η) and ^13^C longitudinal relaxation rate R1 are sufficient to measure the order parameters (S^2^) and characteristic times, describing the internal motions [32]. NOEs of Ly29–43^C,C^ are substantially higher than those of Ly29–43^C-C^, especially on the terminal regions of the peptide, implying the enhanced mobility (Figure 3C). In turn, the detailed analysis of Ly29–43^C-C^ reveals that the peptide is relatively stable. We obtained an almost uniform S^2^ distribution for all the residues (0.7–0.8) in this peptide (Figure 4).

### 3.5. NMR Analysis of Synthetic Analogs of the L. stagnalis AChBP Loop C

**Figure 5:**
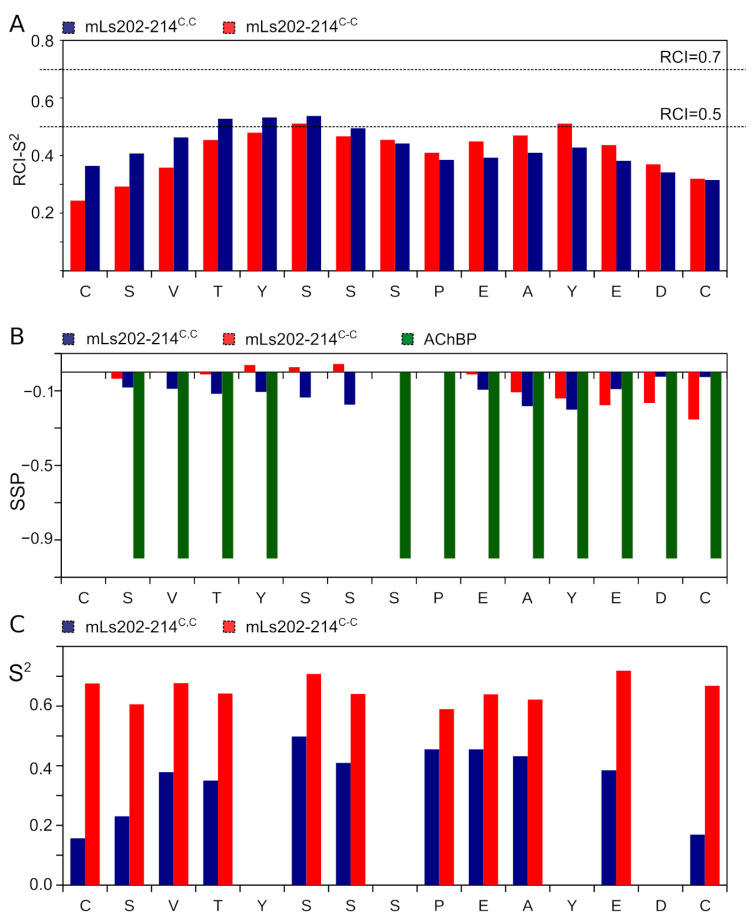
Distributions of RCI-S^2^ (**A**), SSPs (**B**) and order parameters of CαH bonds (**C**) for m*Ls*202–214^C,C^ (blue bars) and m*Ls*202–214^C-C^ (red bars). Negative values of SSP correspond to the β-sheet or extended conformation. SSPs for the secondary structure of the 202–214 region of full-length *L. stagnalis* AChBP (PDB ID: 1UX2) is shown by green bars on panel **B** for comparison.

Using a similar approach, we analyzed the properties of mutated cyclic and linear *L. stagnalis* AChBP loop C fragments—m*Ls*202–214^C-C^ and m*Ls*202–214^C,C^. Similarly to the Lynx1 loop, the linear peptide appeared less compact: (282 ± 5)·10^−12^ m^2^/s and 1.7 kDa vs. (256 ± 3)·10^−12^ m^2^/s and 2.2 kDa. The linear peptide m*Ls*202–214^C,C^ is also much more mobile than cyclic m*Ls*202–214^C-C^, according to the S^2^ magnitudes (Figure 5C). On the other hand, unlike Lynx1, the effect of cyclization on the structure of the loop C peptides appeared rather subtle. RCI-S^2^ profiles of m*Ls*202–214^C,C^ and m*Ls*202–214^C-C^ are highly similar and hardly approach 0.5, suggesting the dynamic behavior of two peptides. Both m*Ls*202–214^C,C^ and m*Ls*202–214^C-C^ are mostly unstructured with SSPs in the range −0.2: +0.1 (Figure 5A,B). The cyclic peptide reveals a higher but still subtle propensity for the extended conformation. No long-range NOESY cross-peaks were found in NMR spectra, in agreement with all data above. Thus, none of the peptides reveal the secondary structure of a parent protein (Figure 5B) found in the respective X-ray structures of the parent AChBP.

## 4. Discussion

**Figure 6:**
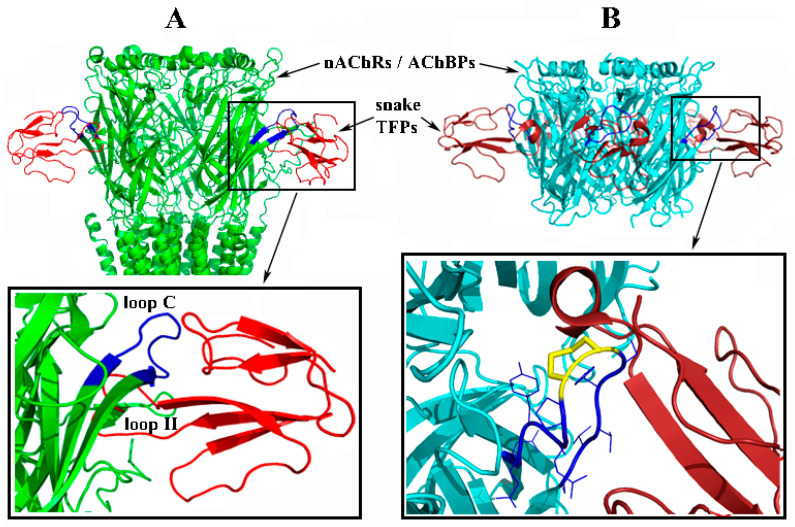
Cartoon-presentations of high resolved structures of nAChRs/AChBPs complexed with snake TFPs. (**A**) A portion (ligand-binding domains with top part of trans-membrane α-helixes) from cryo-EM structure of a native *Torpedo* electric ray nAChR (green) in complex with α-Bgt from *Bungarus multicinctus* (red) (PDB ID: 6UWZ). Loops C of the two α-subunits of the receptor are marked in blue. The inset shows the location of loop C and the central loop II of the toxin at an enlarged scale. (**B**) X-ray structure of AChBP from *L. stagnalis* mollusk (cyan) in complex with α-cobratoxin from *Naja kaouthia* (ruby) (PDB ID: 1YI5). All five loops C of the protein are marked in blue. The inset shows at an enlarged scale the location of the toxin central loop II and AChBP loop C (amino acid residues 202–214) with the side chains (vicinal disulfide CC^207−208^ is marked in yellow).

The present communication is devoted to the analysis of the TFPs interactions with nAChRs, but covers it at a “lower level”, namely by studying the interactions between the TFP fragments and the whole-size nAChRs and, on the other hand, the associations between the nAChR binding site synthetic fragments or their models with the whole-size TFPs. To date, numerous biochemical studies have demonstrated that the main “beneficiaries” of high affinity of the most well-known snake α-neurotoxin TFPs towards distinct nAChR subtypes are the toxin central loop II and the loop C of the receptor α-subunit, respectively. Direct confirmation of this was obtained recently by a cryo-electron microscopy high resolution structure of the α-Bgt complex with the *Torpedo* nAChR [18] (Figure 6A), while earlier the relevant information was based on the X-ray structures of α-neurotoxins in complexes with AChBPs [20,23] (Figure 6B) and with the monomeric ligand-binding domains of the α1 and α9 subunits [19,24].

These X-ray and cryo-electron microscopy data are extremely important both for fundamental research and for practical purposes. In particular, our recent paper [10] was initiated by the well-known facts that synthetic fragments of the central loop II of some snake venom TFPs possess a partial activity of the starting neurotoxins against nAChRs, especially when their structure is stabilized by the two disulfide-joined Cys residues grafted at the N-and C-termini (conventionally such peptides can be named as “cyclized”) (see, for example review [7]). The novelty of that communication was the synthesis and analysis of binding of such fragments of those TFPs of the Ly6 family which are endogenous regulators of the nAChRs in human and other organisms. Interestingly, against the *T. californica* nAChR, the fragment of the water-soluble Lynx1 revealed the activity quite close to that of the starting protein. Another our paper [38] was devoted to a short combinatorial peptide HAP which is homologous to the nAChR loop C and binds α-Bgt with a surprisingly high affinity (2–4 nM), which is virtually the same as for α-Bgt binding to the whole-size nAChRs [15]. Kudryavtsev et al. [38] confirmed this result with a considerably extended arsenal of methods for the analysis of HAP binding. After these publications we had several questions: what determines the activity of the synthetic fragments of Lynx1 and of loop C models, what is the real structure of these peptides, how close they are to the conformation of their parent ligands and receptors and what is the role of disulfides?

To answer these questions, we synthesized an additional set of fragments. Keeping in mind that the disulfide introduction at the peptide termini of the TFP loop II in several cases increased the affinity for the nAChRs, we decided to apply such an approach to the synthetic receptor models (HAP and fragment of loop C from the *L. stagnalis* AChBP). That is why we synthesized the analogs of fragments of the Lynx1 central loop II, the loop C from *L. stagnalis* AChBP and the high-affinity peptide HAP, which spatial structures were stabilized by a disulfide bond formed between the additional cysteine residues introduced at the N- and C-termini of these fragments: Ly29-–43^C-C^, m*Ls*202–214^C-C^ and HAP^C-C^, respectively. The control samples in the biological tests were their linear (non-oxidized) forms—Ly29–43^C,C^, m*Ls*202–214^C,C^ and HAP^C,C^, as well as two original fragments without flanking cysteines—Ly29–43 and HAP. In the case of the loop C fragment, its mutant form was also used with the replacement of vicinal cysteines with serines to avoid the formation of mis-folded Cys-isomers. In addition, it was previously shown that the presence of vicinal disulfide in the corresponding fragments of the nAChR α1-subunit does not strongly affect the binding of snake venom neurotoxins [39,40]. The natural loop C with vicinal cysteines closed in disulfide (*Ls*202–214^C-C^) or in linear form (*Ls*202–214^C,C^) were characterized for their activity as well. The chemical structures of all synthesized peptides were confirmed by mass spectrometry (Table 1) and their biological activities were evaluated by competitive radioligand assay on *T. californica* nAChR.

The biological activity of fragments of several other Ly6 proteins, namely, their ability to compete with radioactive α-Bgt for binding to different nAChRs and such their models as AChBPs and ligand-binding domains of individual subunits, as well as the ability of these fragments to inhibit currents in certain nAChR subtypes, was described by us in detail earlier [10]. The present study confirmed the micromolar affinity of fragments of the central loop II of the Lynx1 protein towards the *T. californica* nAChR (IC_50_ values were in the range 1.1–7.9 μM), showing that the inhibitory activity was higher than that of the full-size protein for the same receptor or AChBPs from *L. stagnalis* and *Aplysia californica* [25] and does not strongly depend on whether the fragment is linear or cyclized (Figure 1A).

These AChBPs, which are structural homologues of ligand-binding domains of nAChRs and often reproduce the nAChR pharmacological profiles, are still actively used in the X-ray crystallography in complexes with various cholinergic ligands. After these structural studies, it became possible to clarify the mechanism of nAChRs functioning and the detailed spatial structure of their orthosteric binding sites, although information about the key amino acid residues of the receptor binding site already existed. In particular, the important role of loop C of the α-subunits of nAChRs was known, which was used for the combinatorial design of a short 13-member peptide that mimics this fragment of the *T. californica* receptor. This peptide was called a high-affinity peptide (HAP) because it showed a nanomolar affinity for α-Bgt, a blocker of distinct nAChR subtypes [15], and it mimicked the structure of the receptor loop C in complex with this toxin [16]. Interestingly, the HAP sequence combines the amino acid residues of the α-subunit of *T. californica* nAChR and *L. stagnalis* AChBP.

In the present study, for the first time, we intended to evaluate the influence of spatial structure on the activity of the *L. stagnalis* AChBP loop C fragment in linear and cyclized forms and to compare it with those for HAP and its analogs. The introduction of additional flanking cysteines into the HAP sequence and their cyclization did not affect the high ability of this peptide to bind ^125^I-α-Bgt, thus preventing association of this radioligand with *T. californica* nAChR (Figure 1B). To our surprise, neither linear nor cyclized forms of loop C where two vicinal Cys were substituted for Ser (as in HAP) could bind the radioligand. This result cannot be explained by the mutation introduced into the loop C structure, since a similar lack of ability to bind ^125^I-α-Bgt was detected for the fragment with vicinal disulfide (Figure 1B).

This difference in biological activity between the native loop C and its combinatorial variant—HAP—may be due to the difference in their spatial structure. Therefore, we estimated the content of secondary structure elements by circular dichroism for all synthesized peptides. The application of CD to small peptide molecules to characterize their secondary structure may raise questions, but there is a sufficient number of examples in the literature when a disruption, for example, of the disulfide bond in a small peptide leads to a noticeable change in the CD spectrum [41,42,43]. We expected that the introduction of two Cys at the ends of the peptide termini with the subsequent closure of the disulfide could affect the content of secondary structure elements and possibly lead to stabilization of the spatial structure. However, in our case the CD measurements did not reveal substantial difference between the linear and cyclized forms (Figure 2, Table 2).

**Figure 7:**
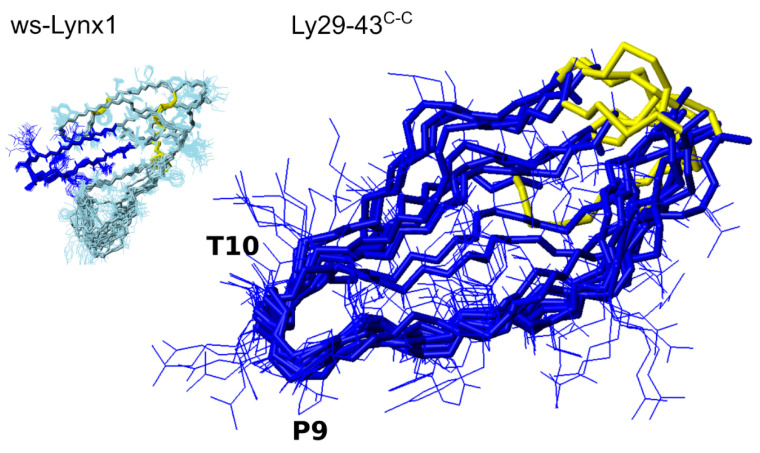
At top shown is the structure of ws-Lynx1 [26] with the loop II highlighted in dark blue. At bottom, the 10 best NMR structures of Ly29–43^C-C^ out of 100 calculated are superimposed over the backbone atoms of residues 3–14. Disulfide bridges are highlighted in yellow.

In order to obtain more precise information about the spatial structure and stability of peptides under investigation, we applied the solution NMR approaches to four objects: Ly29–43^C,C^, Ly29–43^C-C^, m*Ls*202–214^C,C^ and m*Ls*202–214^C-C^. All the peptides under investigation were partially disordered, thus we had to utilize the secondary structure propensity techniques and NMR relaxation analysis to describe the structural ensembles present in solution. Among all the peptides, the ws-Lynx1 fragment fixed by a disulfide Ly29–43^C-C^ was the most stable, with SSP up to 50% in several regions, which allowed us to apply the conventional NMR approaches to solve the structure of the peptide in solution (Figure 7). Although being in general more mobile than the respective loop in the ws-Lynx1, the Ly29–43^C–C^ has a very similar conformation to the respective part of ws-Lynx1.

On the contrary, the linear peptide Ly29–43^C,C^ was highly flexible and disordered. However, it revealed even higher nAChR-binding activity as compared to the cyclized variant (Figure 1A). This is an unexpected result, implying the conformational selection mechanism of nAChR binding. In addition, it is impossible to ignore the presence in the linear form of two active sulfhydryl groups, which can form additional hydrogen bonds in the receptor binding site, or even participate in disulfide exchange with vicinal cysteines of the C-loop of the receptor α-subunit thereby increasing the affinity.

A markedly different result was obtained for the synthetic *L. stagnalis* AChBP loop C fragments in linear or cyclized forms (m*Ls*202–214^C,C^, m*Ls*202–214^C-C^). According to ^1^H-NMR analysis (see Figure 5) none of these peptides had a fixed three-dimensional structure. We had already mentioned that the X-ray structures of complexes of various agonists and antagonists with the AChBPs, where the loop C of the latter was always playing a major role and was used to model the interactions with the whole-size nAChRs. What concerns the interactions with α-neurotoxins, it was earlier demonstrated for some fragments covering the loop C of the α1 nAChR subunit and containing vicinal cysteines that toxin binding was not extremely sensitive to the state (free or oxidized) of these cysteines [39,40]. In the present communication the AChBP loop C fragments were synthesized and tested in their interaction with α-Bgt for the first time and we still cannot explain why they were inactive and why the stabilization of the loop C spatial structure by disulfide closure did not give any promising results.

Summarizing, we have shown the effect of disulfide on the structure and stability of peptide fragments in one case (for loop II of Lynx1), sometimes (for loop C of AChBP and for HAP) it is absent, while the structuring of the peptides in solution does not necessarily correlate with their activity: the most active was the linear peptide Ly29-43^C,C^, that contrasts with the least active but the most structured ligand—the full-size ws-Lynx1. Anyway, because for various Ly6 proteins such as ws-Lynx1 or SLURP-1 are carried out versatile tests against cancer and neurodegenerative diseases [44,45] we believe that a set of obtained and characterized synthetic fragments can also be useful.

## 5. Conclusions

The main result is that a set of the synthetic fragments of the loop II of Ly6 TFP was prepared, including those where the additional Cys residues were introduced at the N- and C-termini and left free or oxidized to the disulfide. The inhibitory activity of these peptides was tested against *Torpedo* nAChR and some linear forms or those “cyclized“ by a disulfide were found to be more active than the ws-Lynx1. By ^1^H-NMR we found that the disulfide-fixed Lynx1 fragment has a three-dimensional structure very similar to the loop II within the whole-size ws-Lynx1. We also tried to apply such “cyclization” approach to the models of the nAChR binding sites, namely to AChBP loop C and HAP. The latter retained its binding activity in linear and cyclized forms, in contrast with the loop C which did not reveal for both forms either the binding capacities or an ordered secondary structure.

## Figures and Tables

**Table 1:** Synthetic fragments of Lynx1 central loop, *L. stagnalis* AChBP loop C and HAP with their abbreviations and measured molecular masses.

№	Protein Fragment	Sequence	Theoretical Mass/Measured Mass, Da	Abbreviation
(1)	Human Lynx1 fragment 29–43 (Loop II).	TTRTYYTPTRMKVSK	1833.0/1832.9	Ly29–43
(2)	Human Lynx1 fragment 29–43 with cysteines at N- and C- termini, linear form.	CTTRTYYTPTRMKVSKC	2039.0/2039.0	Ly29–43^C,C^
(3)	Human Lynx1 fragment 29–43 with cysteines at N- and C-termini, cyclized form.	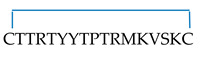	2037.0/2037.1	Ly29–43^C-C^
(4)	*L. stagnalis* AChBP fragment 202–214 (Loop C), linear form.	SVTYSCCPEAYED	1466.5/1466.4	*Ls*202–214^C,C^
(5)	*L. stagnalis* AChBP fragment 202–214 (Loop C), cyclized form.	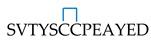	1464.5/1464.6	*Ls*202–214^C-C^
(6)	*L. stagnalis* AChBP fragment 202–214 with cysteines 207–208 mutated to serines and cysteines added at N- and C- termini, linear form.	CSVTYSSSPEAYEDC	1640.6/1640.6	m*Ls*202–214^C,C^
(7)	*L. stagnalis* AChBP fragment 202–214 with cysteines 207–208 mutated to serines and cysteines added at N- and C- termini, cyclized form.	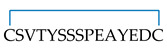	1638.6/1638.6	m*Ls*202–214^C-C^
(8)	Combinatorial high-affinity peptide HAP.	WRYYESSLLPYPD	1688.8/1688.7	HAP
(9)	Combinatorial high-affinity peptide HAP with cysteines at N- and C- termini, linear form.	CWRYYESSLLPYPDC	1894.8/1894.8	HAP^C^^,C^
(10)	Combinatorial high-affinity peptide HAP with cysteines at N- and C- termini, cyclized form.	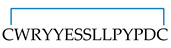	1892.8/1892.9	HAP^C^^-C^

**Table 2:** Secondary structure of peptides (1)–(10) according to CD spectrum analysis.

Peptide	α-Helix, %	β-Structure, %	β-Turn, %	Random, %	NRMSD ^1^
(1)	5.2	31.5	22.6	40.7	0.02
(2)	5.1	32.6	22.5	39.8	0.02
(3)	4.1	38.5	22.5	34.9	0.03
(4)	5.9	32.4	23.4	38.2	0.02
(5)	6.1	30.9	23.7	39.2	0.02
(6)	6.4	36.1	23.0	34.5	0.03
(7)	5.9	33.6	22.4	38.0	0.02
(8)	3.3	39.5	22.6	34.6	0.03
(9)	3.9	38.5	22.3	35.3	0.03
(10)	3.7	39.9	21.7	34.7	0.05

^1^ According to [36], the analysis is considered to be reliable if NRMSD is < 0.1.

## Supplementary Materials

The following are available online at https://www.mdpi.com/2218-273X/11/1/1/s1, File S1: The data for the spatial structure of Ly29–43^C-C^.
